# The Effect of Stabilization Procedures on Sports Discipline and Performance Level in Non-Elite Athletes after Acute Syndesmotic Injury: A Prospective Randomized Trial

**DOI:** 10.3390/jcm11154609

**Published:** 2022-08-08

**Authors:** Christian Colcuc, Dirk Wähnert, Florian J. Raimann, Thomas Stein, Sanjay Weber-Spickschen, Reinhard Hoffmann, Sebastian Fischer

**Affiliations:** 1 Department of Trauma and Orthopedic Surgery, Protestant Hospital of Bethel Foundation, University Hospital OWL of Bielefeld University, Campus Bielefeld Bethel, Burgsteig 13, 33617 Bielefeld, Germany; 2 Department of Aneasthesiology, Intensive Care Medicine and Pain Therapy, University Hospital Frankfurt, Goethe University, Theodor-Stern-Kai 7, 60590 Frankfurt, Germany; 3 SPORTHOLOGICUM® Frankfurt Am Main, Siesmayerstraße 44, 60323 Frankfurt, Germany; 4 Department of Sports Medicine, Goethe University Frankfurt, Ginnheimer Landstraße 39, 60487 Frankfurt, Germany; 5 Traum and Sports Department, Peine Hospital, 31226 Peine, Germany; 6 Department for Trauma and Orthopaedic Surgery Berufsgenossenschaftliche, Unfallklinik Frankfurt am Main, 60389 Frankfurt, Germany; 7 Department of Foot and Ankle Surgery Berufsgenossenschaftliche, Unfallklinik Frankfurt am Main, 60389 Frankfurt, Germany

**Keywords:** ankle syndesmosis, return to sports, syndesmotic screw, knotless suture button device

## Abstract

Background: Suture button devices for tibiofibular syndesmosis injuries provide semirigid dynamic stabilization. The effect of stabilization procedures on sports discipline and performance level in non-elite athletes after acute syndesmotic injury has not been clarified in sports medicine research to date. Methods: A total of 47 of 56 eligible patients were analyzed and completed the 1-year follow-up. The average age was 35.5 years (range, 18–60 years). The screw fixation and knotless suture button groups comprised 26 and 21 patients, respectively. Nine patients were lost to follow-up. Patients underwent clinical and radiological evaluations preoperatively and twice during the 1-year postoperative follow-up. Function was measured using the FADI sports scale, the FAAM sports module, and a visual analogue scale for pain and function in sports. Questionnaires were completed to assess preoperative and postoperative sports levels and to evaluate the sports discipline. Results: All scores increased during the follow-up, but no significant differences were found in the FADI score, the FAAM sports module score and or the VAS score for pain and function during sport (*p* ≤ 0.05). Using Spearman’s rank correlation coefficient, we found no significant correlation between the groups for age, injury mechanism, or body mass index. Differences were identified in sports discipline and performance level between the groups during the follow-up period. Conclusion: No statistically significant differences could be demonstrated between the two stabilization methods in terms of return to previous sport level and return to the original sport discipline, so both procedures can be regarded as equivalent at present.

## 1. Introduction

Syndesmosis injuries, or high ankle sprains, represent up to 25% of all ankle sprains [1,2,3] and are associated with 10% of ankle fractures [4]. As part of the injury, there is a pronation–eversion movement in the talus in the ankle mortise, combined with an ankle dorsiflexion. The fibula performs a lateral movement from anterior to posterior during the accompanying external rotation. The medial malleolus provides a fixed bearing. Isolated rupture or rupture of the syndesmosis combined with fractures may result from this [5].

Participants in high-contact sports such as football, lacrosse, and hockey are thought to be more susceptible to ankle syndesmosis injury [2,6,7], as these sports may involve extremely fast rotations and a sudden, powerful external rotation of the foot with the widening of the ankle joint. Due to the partial weight-bearing phase of at least 6 weeks and a possible second operation with removal of the screw, the rehabilitation period is longer than for other ankle sprains.

Undiagnosed or incorrectly treated syndesmosis injuries can lead to a prolonged recovery time and worsened athletic performance [1,7,8,9]. An essential prerequisite for returning to sporting activity and performance is an intact and stably healed syndesmosis complex after syndesmosis injury. For elite athletes, as well as non-elite athletes, returning to the previous sports discipline and performance level after an injury is a key issue.

Therefore, the identification of the most effective treatment is challenging, and potential treatments are still being investigated.

Syndesmosis injuries are frequently treated with a metallic screw or with a semi-rigid knotless suture button device.

The advantages of screw implantation are that it is inexpensive and quick to use intraoperatively. The disadvantage is that screw fractures may occur, and hardware removal is usually performed as a second surgical procedure. Synostosis and ossification are described in the literature for some cases [10].

In addition to the faster rehabilitation time, the suture button implantation does not require further surgery with removal of the implant. However, the implant costs are higher than they are with the screw. The described problems with knot irritation could be solved with the second generation of the suture button.

A previous clinical study has shown that the use of the suture button repair in patients with distal tibiofibular syndesmosis rupture was advantageous in highly active patients because it allowed for an earlier return to sports activity [11].

Therefore, the hypothesis of this subgroup-analyzed randomized controlled trial is that also, with regard to the return to the previous sport discipline and level, knotless suture button fixation after unstable syndesmosis injury is beneficial for non-elite athletes.

To the best of our knowledge, the effect of stabilization procedures on sports discipline and performance level in non-elite athletes after acute syndesmotic injury in sports medicine research has not yet been clarified.

## 2. Materials and Methods

### 2.1. Patient Selection

This single-center prospective randomized controlled trial was conducted to assess the preoperative and postoperative sports type and sports level in patients with ankle injuries involving distal tibiofibular syndesmosis.

Patients with acute isolated syndesmosis disruption, as well as patients with additional fractures of the lateral ankle, were identified from 2012 to 2015.

The inclusion criteria were as follows: age from 18 to 60 years, sports at the non-elite level, and the presence of syndesmosis injury. The exclusion criteria were as follows: no sports activity; contralateral ankle symptoms or surgery; a history of previous ankle surgery; rheumatologic, neuromuscular or autoimmune disease; lack of compliance.

The injury was verified by standard radiograph in mortise view, lateral projection under weightbearing and clinical stress tests. In case of a suspected isolated syndesmosis injury, an MRI examination was performed as an additive measure.

Only in cases of suspected concomitant fracture were radiographs taken without weightbearing, and the instability was verified intraoperatively.

The clinical stress examination was performed with the dorsiflexion–external rotation test and the squeeze test under fluoroscopy and plain radiographs.

If plain or stress examination under fluoroscopy demonstrated a >2-mm side-to-side difference in the tibiotalar or tibiofibular clear space compared with the contralateral uninjured limb, the syndesmosis injury was defined as unstable and the indication for stabilization was given [12].

All patients provided written informed consent for participation in this study. Approval was obtained from the local ethics board, and the Consort guidelines were followed for this study (http://www.consort-statement.org, 1 October 2012).

### 2.2. Randomization Procedure

Patients with associated preoperative or intraoperative evidence of syndesmotic disruption were identified. A randomization list was created by a biostatistician. The randomization process involved randomly changing block sizes of four and six. The randomized surgical procedure then was placed into numbered, opaque sealed envelopes in a box to ensure concealment. After establishing a diagnosis of an unstable syndesmosis in the operating room, an assistant nurse removed and opened one numbered envelope containing the method of syndesmosis fixation.

Fifty-six consecutive patients who met the study inclusion criteria and consented to participate in the study were randomized via the above-described procedure.

**Figure 1:**
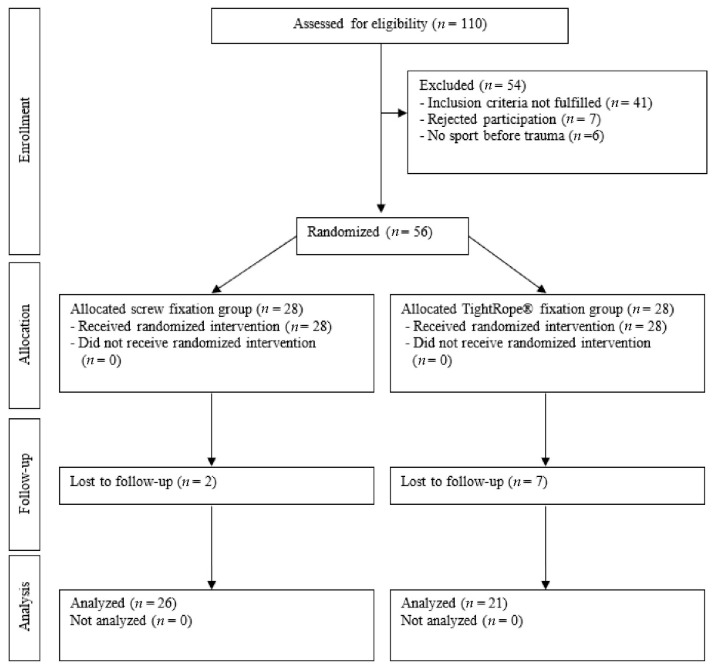
Flow chart for patient selection.

Each group comprised 28 patients who were evaluated (Figure 1).

The average patient age was 35.5 years (range, 18–60 years). The patient demographics are listed in Table 1.

### 2.3. Surgical Technique

The operations were performed by three surgeons who were experienced in treating ankle injuries and familiar with both operation procedures. All fractures were stabilized prior to insertion of the knotless TightRope**^®^** or 3.5-mm transosseous syndesmotic screw and an intraoperative stress examination was performed under fluoroscopy. The reposition was temporarily fixed with a guide wire. The accuracy of the syndesmotic reduction was confirmed using three-dimensional (3D) imaging with the Arcadis Orbic 3D fluoroscope in a side comparison. If malreduction was suggested, the fibula was repositioned, refixed, and rescanned. The syndesmosis was then fixed depending on the outcome of the randomization: either with the knotless TightRope**^®^** device or according to the standard principles of the Association for the Study of Internal Fixation (AO-ASIF) using a 3.5-mm transosseous syndesmotic screw purchasing three cortices. Maisonneuve fractures were fixed with two of the particular implants.

Depending on the fracture morphology, the drillholes were placed approximately 3.5 cm above the ankle joint in the transmalleolar plane. The placement of the knotless TightRope**^®^** button along the medial cortex was confirmed using a C-arm. In all cases, a suturing of the anterior part of the syndesmosis was attempted.

### 2.4. Postoperative Protocol

All patients participated in a rehabilitation program under the supervision of physical therapists. This program involved no weight-bearing, mobilization on crutches for 7 weeks postoperatively, and immobilization in an orthotic boot. All patients followed this program consistently.

On day 2, standard radiograph controls of the ankle in two planes were obtained.

In patients who underwent screw fixation, the screw was removed as an outpatient procedure after 7–8 weeks. Weightbearing was then allowed, and a physiotherapist instructed the patient in performing rehabilitation exercises.

### 2.5. Outcomes Assessment

Outcomes were clinically and radiologically assessed preoperatively, and at 6 and 12 months postoperatively. All patients underwent a clinical examination by an independent clinician who was not blinded to the type of operation. Questionnaires to assess preoperative sports discipline and performance level were completed and documented.

The sports indicated by the patients were divided into three categories: stop-and-go sport (football, tennis, handball, basketball) endurance sport (running, cycling) and individual sport (equestrian sport, fitness sports, swimming).

The sports level was also divided into three levels: competitive sport, recreational sport, and health sport.

The competitive level was made up of patients who had played the sport for a long time and had competition ambitions. They trained more than three times a week.

The recreational sport level refers to patients for whom the primary purpose of the activity is participation, often with the associated goals of improved physical fitness and fun.

Competitive ambitions may be present but tend to be in the background.

Patients without any competition ambitions in sports activity belonged to the category of health sport. This level of sport specifically aims to improve endurance, agility, and strength and support the cardiovascular system.

The time between surgery and the resumption of sport, with reference to the sport discipline and sport level, was documented by a clinician, and radiographs in mortise view, lateral projection, and under weightbearing of the ankle were obtained and assessed for degenerative changes and to rule out instabilities.

A follow-up examination was conducted using the Foot and Ankle Disability Index (FADI) with the sports module and the Foot and Ankle Ability Measure (FAAM) sports module [13].

The scores used are designed to measure foot and ankle function and record different movement patterns and endurance at the specified examination times.

Additionally, a 100 mm visual analogue scale (VAS) for function and pain in sports was employed.

### 2.6. Statistical Analysis

All the analyses were performed by a professional independent statistician using the SPSS software (SPSS Statistics for Windows, version 21.0, IBM Corp., Armonk, NY, USA). The patient demographics were compared between the two groups using mean values and proportions. The scores were compared between the two groups using the Mann–Whitney U test for independent random samples. Mean values were also calculated for the clinical outcome scores, and Student’s *t*-test was used to compare these values within each group and determine statistical significance. A *p* value of ≤0.05 was considered statistically significant. Spearman’s rank correlation test and a bivariate analysis were performed to adjust for potential confounders, such as patient age, body mass index (BMI), and injury type, to identify the variables affecting the clinical outcome scores.

The a priori analysis of required sample sizes, using the Mann–Whitney U test, was performed with an effect size of d = 0.8 (large effect), α = 0.05, and a power (1 − β) of 0.80, giving a minimum sample size of 42 participants (*n* = 21 in each group).

Using Spearman’s rank correlation coefficient, we found no significant correlation between the two groups for age, injury mechanism or BMI.

## 3. Results

A total of 110 patients were eligible for inclusion during the 3-year study period. Forty-seven patients did not fulfill the inclusion criteria and were excluded. A further seven patients declined to participate in the study, so a total of 56 patients were enrolled and evaluated.

Nine patients were lost to follow-up (16%) within 1 year and were thus excluded from the analysis (Table 2).

Overall, 47 patients (26 in the screw fixation group and 21 in the knotless TightRope**^®^** fixation group) with a mean age of 35.5 years were analyzed and completed the 1-year follow-up.

No significant differences in the demographic data and injury patterns were found between the two groups (Table 1).

Additionally, no significant differences were found in the FADI score and the FAAM sports module (Table 3).

The VAS scores improved from the preoperative assessment to the last follow-up. No significant between-group differences were detected during the follow-up period (Table 4).

**Figure 2:**
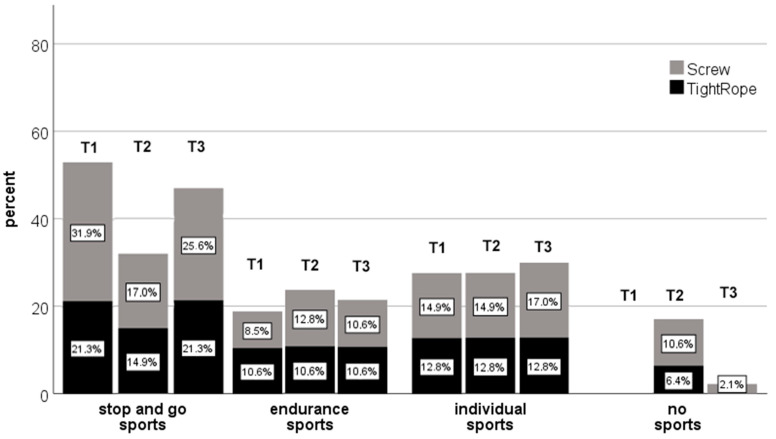
Kind of sport within the two groups before, 6 months and 1 year after syndesmosis Injury.

After 6 months (T2), 6% of the TightRope**^®^** patients and 11% of the screw patients in the stop-and-go sport disciplines were not yet able to resume their sport or switched to another sport discipline with less stress (endurance sport). However, after 12 months, all TightRope**^®^** patients had returned to their stop-and-go sport, whereas 6% of the screw patients had to change their sport permanently due to the injury. In both endurance sport and individual sport, all TightRope**^®^** patients and all screw patients were able to return to their sport discipline after 6 months. Two percent of the screw patients were unable to participate in sports, even after 1 year (Figure 2).

No differences were found between the two groups at the competitive sports level.

**Figure 3:**
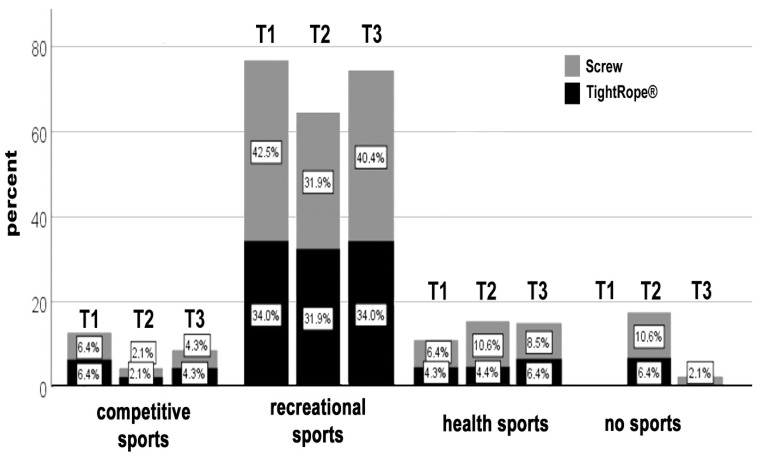
Sports ability and exercise sports level of the screw fixation group and the knotless TightRope^®^ fixation group at the three examination times.

In the recreational sports group, almost all patients treated with the knotless TightRope**^®^** device maintained their sports level after 6 months (T2), but this advantage leveled out after 1 year (T3) (Figure 3).

In contrast, approximately 10% of the screw fixation group patients did not reach their previous level of performance after 6 months.

One patient from each group changed to a lower sports level during the year.

More patients in the screw fixation group were unable to participate in sports at time point T2 compared with the knotless TightRope**^®^** fixation group. Even after 1 year, 2% of patients in the screw fixation group were still unable to participate in sports, whereas all knotless TightRope**^®^** patients were active again. All results showed no statistical significance.

No chronic instabilities, radiographic diastasis in the weightbearing x-ray exposures, wound complications, or infections were seen during the follow-up period.

## 4. Discussion

The most important finding of our study was that there were no statistical differences in the clinical scores used, consisting of FAAM and FADI with their sports modules. There was also no significant difference between the two stabilization methods with regard to the examined return to the previous sport discipline and performance level.

The additionally used 100-mm VAS for function, and pain in sport improved from the first postoperative assessment to the last follow-up, but, again, no significant differences were found between the groups.

However, non-elite athletes treated with the knotless suture button device were able to reach their previous level of performance in recreational sports slightly earlier than the screw fixation group and did not have to change their sports discipline as a result of the injury.

Our hypothesis that the knotless stabilization system offers advantages in terms of time to return to previous sport discipline and performance level compared to screw stabilization in non-elite athletes could not be statistically confirmed.

Our findings of the functional outcomes of the knotless suture button system are consistent with other reports. One study reported a significantly faster return to sports in patients treated with the knotless suture button system [11].

Calder et al. reported the results of 36 professional athletes who underwent surgical stabilization of an unstable isolated syndesmotic injury. All athletes were able to return to play after a mean 65 days and were able to resume their previous sporting activities at the same competitive level [14]. Another study by D’Hooghe et al. evaluated the time to return to play in a cohort of professional male football players following surgical stabilization of isolated unstable syndesmosis [15]. They reported that the time taken to return to team training was 72 ± 28 days and the first official match was played, on average, after 103 ± 28 days. Most injured professional football players (95%) returned to match-play within 6 months of surgery.

In contrast with our study, both studies included only isolated syndesmosis injuries and there was no differentiation between the different sports levels, sports disciplines, and operative fixation methods. Moreover, all patients were male elite level league players of a similar age, fitness level, motivation, and access to therapy and equipment. Therefore, a direct comparison with non-elite athletes in popular sports is not possible.

The use of suture button repair aims to allow for a more physiological motion of the distal fibula with respect to the tibia while maintaining adequate stability and is, therefore, associated with faster rehabilitation and a faster return to sports [11,16]. This might be one reason why patients treated with the knotless suture button device showed a tendency toward a faster return to their previous recreational sports level. Another possible factor influencing the rehabilitation time of the screw group is the second surgical intervention after 7–8 weeks as an outpatient procedure. There is controversy regarding the timing and the fact that screws must be removed by default after healing of the syndesmosis. The clinical practice of this technique is highly variable due to the lack of existing literature [17,18]. A short-term rehabilitation delay can, therefore, be assumed compared with the knotless TightRope**^®^**, but the effect on the time to return to the previous sports discipline and performance level would potentially be less after 6 months.

In the clinical sport scores FAAM and FADI, as in the VAS scales, there were no statistical significances between the groups. This is in line with the current literature. Two studies reported that dynamic devices seem to provide better clinical and radiographic outcomes [19,20]. Grassi et al. and Shimozono et al., in their meta-analysis of randomized controlled trials, pointed out that the dynamic fixation of syndesmotic injuries is able to reduce the number of complications and also improve clinical outcomes compared with static screw fixation [17,21]. These, and other studies analyzed in a review of studies from the last 10 years, underscore the potential advantage of dynamic fixation in syndesmotic injuries, but also show that these differences are not statistically significant [22].

Syndesmotic malreduction is the most important predictor of clinical outcomes [23,24].

As standard radiographic measurements are inaccurate [25] and the position of the fibula in the distal tibiofibular joint is individually variable [26], we performed intraoperative bilateral 3D imaging with the Arcadis Orbic 3D fluoroscope in the present study. No postoperative malreduction occurred, but only bilateral plain radiography was performed to assess postoperative malreduction.

Notwithstanding its limitations, this study contributes significantly to the current literature because this is the first prospective randomized series that evaluates the time taken to return to the previous sports discipline and the level of performance in non-elite athletes and, accordingly, for a large number of athletes in popular sports after acute syndesmotic injuries using the knotless TightRope**^®^** system.

Some limitations of this study need to be considered.

First, the patients had different injury classifications and the surgical procedures were performed by three different surgeons. Therefore, performance bias was present. Furthermore, blinding of the outcome assessor was not possible due to the need for a second operative procedure for routine screw removal.

Additionally, follow-up assessments were only performed 6 and 12 months postoperatively. In theory, significant differences could have occurred between the times of the investigation.

No postoperative computed tomography scans were taken to evaluate malreduction, so we could only assess syndesmosis reduction using plain radiographs and intraoperative 3D scans.

Due to the low number of cases in the subgroup analysis, only a descriptive presentation with a clinical tendency can be made, but no statistical significance can be derived.

## 5. Conclusions

No statistically significant differences could be demonstrated between the two stabilization methods in terms of return to the previous sport level and return to the original sport discipline.

However, the TightRope group showed a tendency to return to their previous sport discipline and performance level more quickly in sports with stop-and-go components and in sports at the recreational sport level.

Nevertheless, a clear treatment recommendation for a stabilization procedure cannot be derived from these data, so both procedures can be regarded as equivalent at present.

## Figures and Tables

**Table 1:** Patient demographics and injury patterns.

	Screw (*n* = 26)	TightRope (*n* = 21)
**Sex**		
Female, *n* (%)	5 (19)	5 (24)
Male, *n* (%)	21 (81)	16 (76)
**Age**, y, median (range)	39 (18–60)	32 (18–60)
**Affected side**		
Right, *n* (%)	11 (42)	12 (57)
Left, *n* (%)	15 (58)	9 (43)
**Mechanism of injury**		
Private, *n* (%)	8 (31)	11 (52)
Traffic, *n* (%)	1 (4)	0 (0)
Work, *n* (%)	9 (35)	3 (14)
Sports, *n* (%)	8 (31)	7 (33)
**Classification**		
Weber B, *n* (%)	4 (15)	4 (19)
Weber C, *n* (%)	4 (15)	2 (10)
Isolated, *n* (%)	11 (42)	11 (52)
Maisonneuve, *n* (%)	7 (27)	4 (19)

Data are presented as *n* (%), median (range).

**Table 2:** Reasons for loss to follow-up.

Patient Number	Screw	TightRope^®^
1		Left the country
2	Unable to contact	
3		Unable to contact
4	Left the country	
5		Left the country
6		Withdrawal of participation
7		Withdrawal of participation
8		Unable to contact
9		Unable to contact

**Table 3:** Foot and Ankle Disability Index (FADI) ^a^ Sports Module.

	Presurgery (T1) ^a^	6 Months (T2) ^a^	12 Months (T3) ^a^	*p* Values ^b^	95% CI ^c^
FADI Sports					
Screw	100 ± 0	83 ± 22	86 ± 20	pT1 = n.s.	T1 = 97–100
TightRope^®^	97 ± 7	77 ± 23	87 ± 16	pT2 = n.s.	T2 = 74–87
				pT3 = n.s.	T3 = 81–91
FAAM Sports					
Screw	100 ± 1	80 ± 26	85 ± 18	pT1 = n.s.	T1 = 98–100
TightRope^®^	98 ± 6	79 ± 21	89 ± 14	pT2 = n.s.	T2 = 73–87
				pT3 = n.s.	T3 = 82–91

^a^ Scores shown as mean ± standard deviation. FADI, Foot and Ankle Disability Index Sports Module; FAAM, Foot and Ankle Ability Measure Sports Subscale; ^b^ Mann–Whitney-U-Test; ^c^ Hodges–Lehman Test. FADI, Foot and Ankle Disability Index sports module; FAAM, Foot and Ankle Ability Measure sports subscale; CI, confidence interval.

**Table 4:** VAS Score Pain and Function during sports.

	Presurgery (T1) ^a^		6 Months (T2) ^a^		12 Months (T3) ^a^		*p*-Values ^b^
	Screw	TightRope^®^	Screw	TightRope^®^	Screw	TightRope^®^	
Pain sports	0 ± 0	0 ± 1	1 ± 2	3 ± 3	1 ± 2	2 ± 2	pT1 = 0.194
							pT2 = 0.107
							pT3 = 0.519
Function sports	0 ± 1	0 ± 1	2 ± 2	3 ± 3	2 ± 2	2 ± 2	pT1 = 0.878
							pT2 = 0.194
							pT3 = 0.608

^a^ Scores are shown as mean ± standard deviation. ^b^ Mann–Whitney U test.

## Data Availability

All the data intended for publication are included in the manuscript.
